# Supplementary material for: Effect of Calcination Temperatures on Surface Properties of Spinel ZnAl2O4 Prepared via the Polymeric Citrate Complex Method—Catalytic Performance in Glycerolysis of Urea
Source: Nanomaterials (Basel). 2023 Jun 21;13(13):1901. doi: 10.3390/nano13131901 (PMC10343499; doi:10.3390/nano13131901)
Supplement: Supplementary file 1 [file nanomaterials-13-01901-s001.zip › nanomaterials-2423503-supplementary.pdf]

Supplementary Materials

Effect of calcination temperature to surface properties of spinel $ZnAl_2O_4$ prepared by polymeric citrate complex method – catalytic performance in glycerolysis of urea.

Ngoc Nhiem Pham[1], Huy Nguyen-Phu[2], Eun Woo Shin[1,*]

[1] *School of Chemical Engineering, University of Ulsan Daehakro 93, Nam-gu, Ulsan 44610, South Korea.*

[2] *Department of Chemical and Biomolecular Engineering, Seoul National University of Science and Technology, Seoul 01811.*

**Corresponding author: ewshin@ulsan.ac.kr, Tel.: +82-52-259-2253*

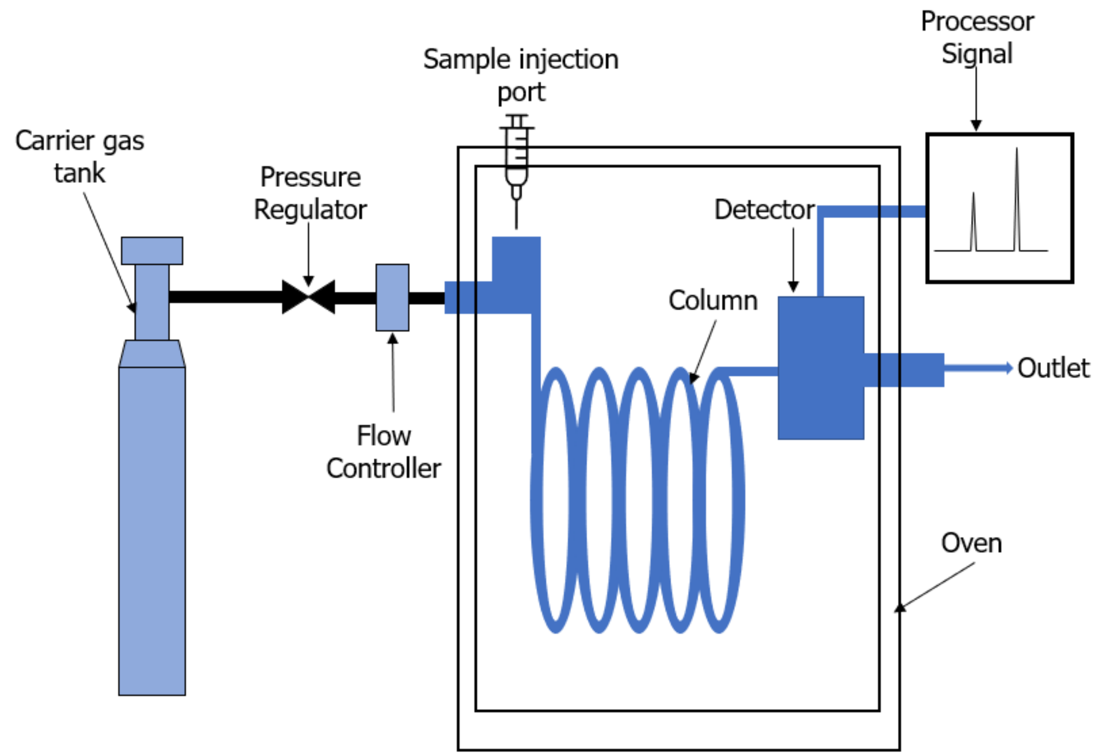

Gas chromatographic analysis conditions:

- Detector type: FID
- Column temperature: 240°C
- Carrier gas: Helium (He)
- Flow rate of carrier gas : 2.1 mL/min

Figure S1. A schematic diagram of the catalyst activity test device with the gas chromatographic analysis conditions.

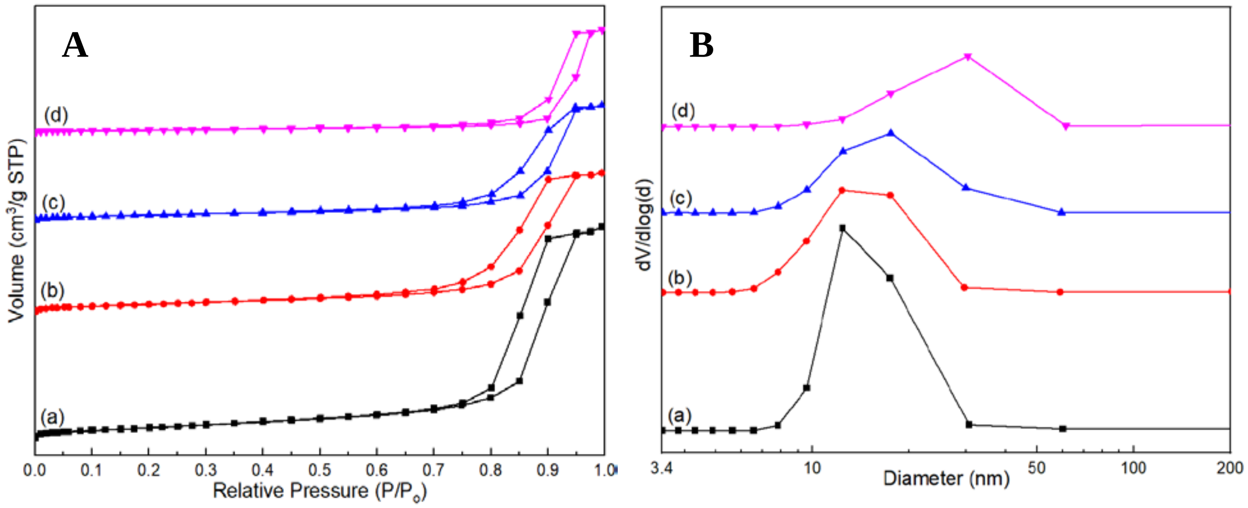

Figure S2. N_2 adsorption-desorption isotherms (A) and pore size distribution curves (B): (a) $ZnAl_2O_4$-550, (b) $ZnAl_2O_4$-650, (c) $ZnAl_2O_4$-750, (d) $ZnAl_2O_4$-850.

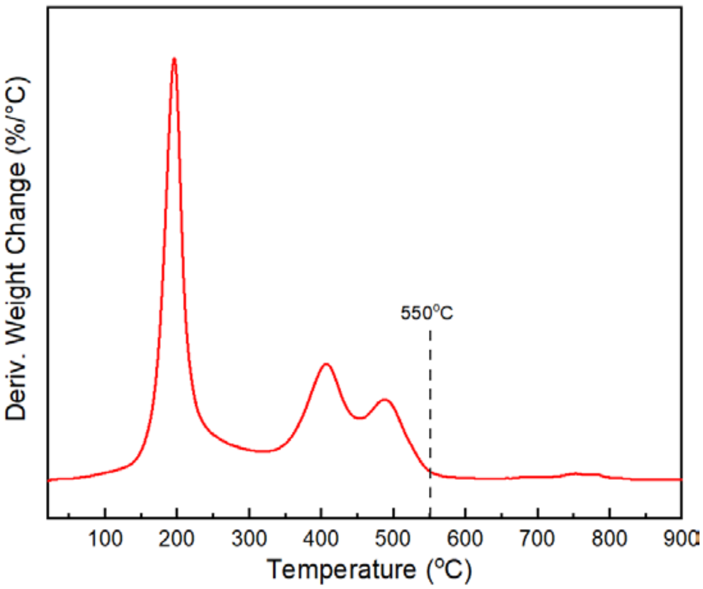

Figure S3. DTGA curves of $ZnAl_2O_4$ xerogel precursor prepared polymeric citrate complex method.

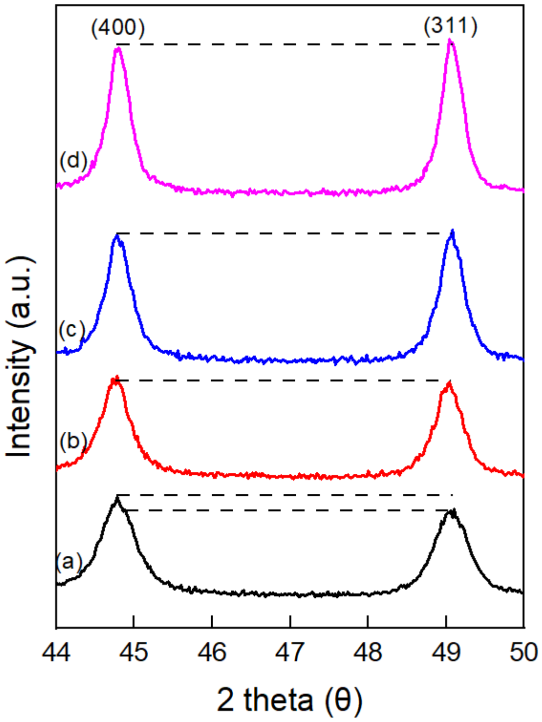

Figure S4. Enlarged XRD patterns of fresh catalysts: (a) $ZnAl_2O_4$-550, (b) $ZnAl_2O_4$-650, (c) $ZnAl_2O_4$-750, (d) $ZnAl_2O_4$-850.

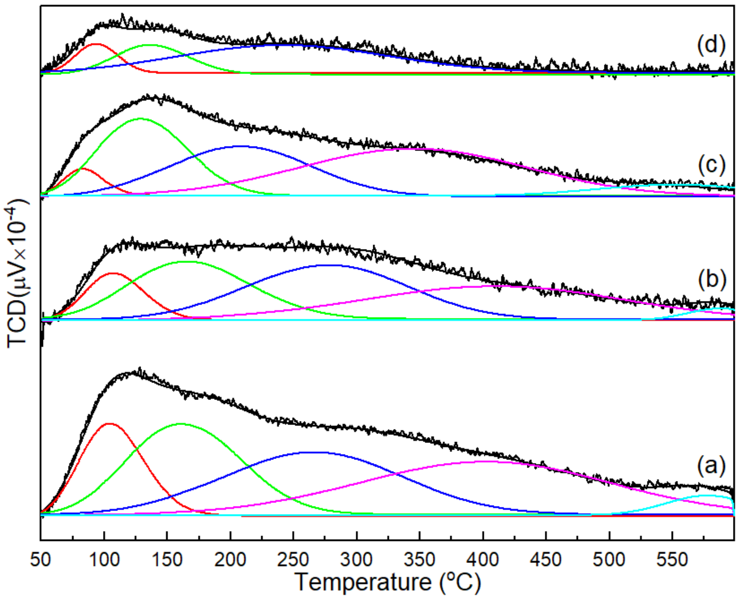

Figure S5. NH_3 profiles of fresh catalysts: (a) $ZnAl_2O_4$-550, (b) $ZnAl_2O_4$-650, (c) $ZnAl_2O_4$-750, (d) $ZnAl_2O_4$-850.

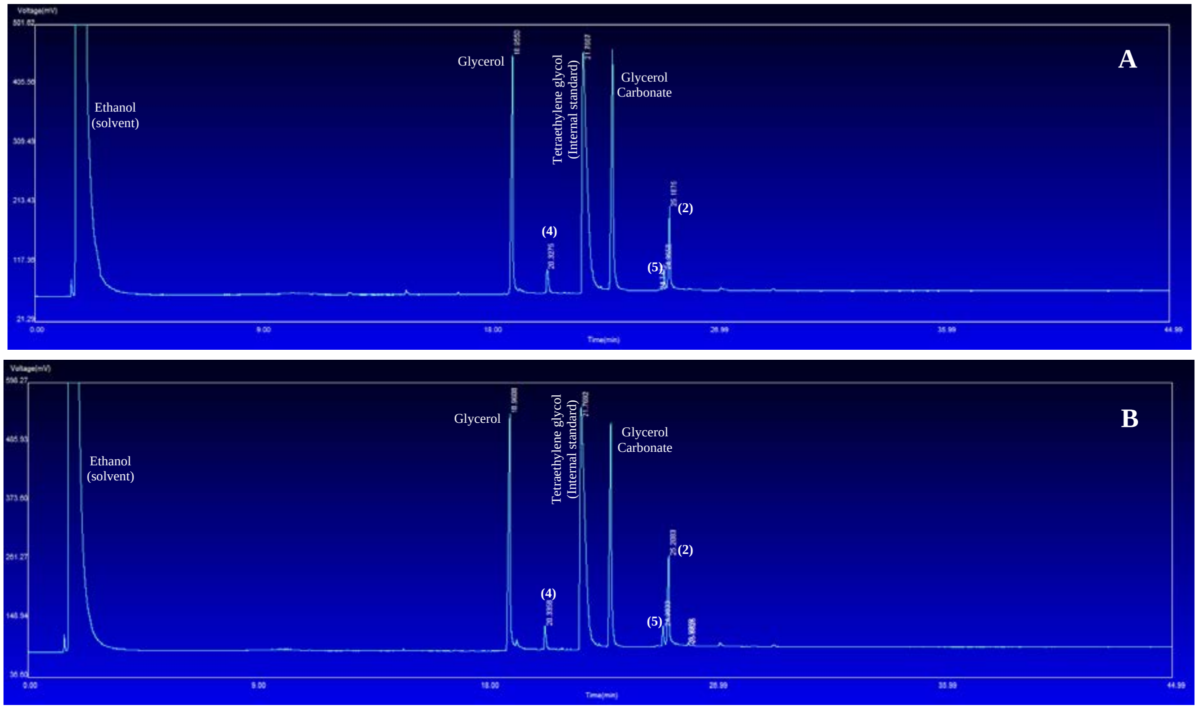

Figure S6. Typical gas chromatograms of $ZnAl_2O_4$-550 (A) and $ZnAl_2O_4$-650 (B). (2): 2,3-dihydroxypropyl carbamate, (4): 4-(hydroxymethyl) oxazolidin-2-one, and (5): (2-oxo-1,3-dioxolan-4-yl) methyl carbamate.

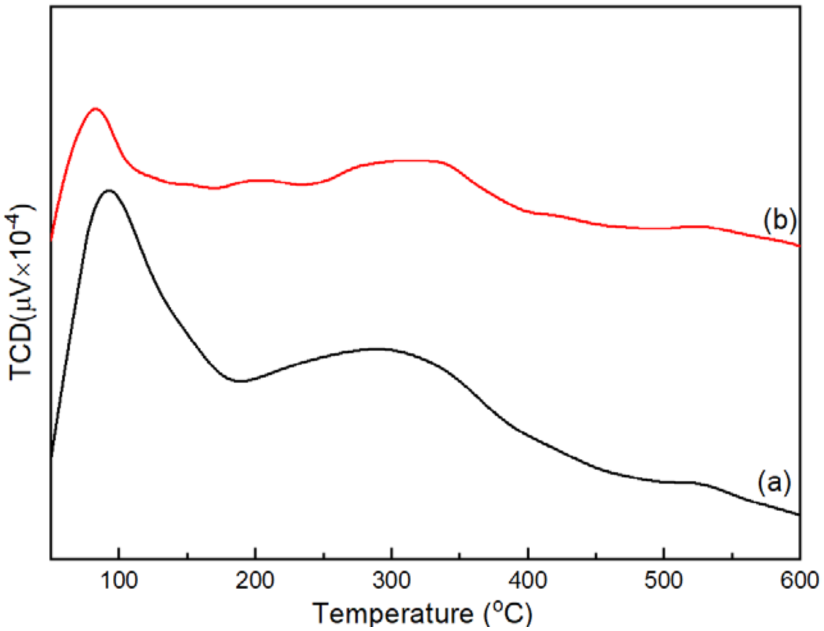

Figure S7. CO_2 profiles of fresh catalysts: (a) $ZnAl_2O_4$-550, (b) $ZnAl_2O_4$-650.

Table S1. The initial amount of each reactant; the amount of glycerol and products after reaction and carbon balance.

Catalyst	Initial Amount of Glycerol (mmol)	Initial Amount of Urea (mmol)	Amount of Glycerol after reaction (mol)	Amount of products (mmol)				Carbon balance (%)
				GC	(2)	(4)	(5)	
$ZnAl_2O_4$-550	200	203	143	66	17	5.5	4	99.8
$ZnAl_2O_4$-650	203	203	144	60	23	6.6	4.2	99.9
